# Synergistic anti-cancer effect of sodium pentaborate pentahydrate, curcumin and piperine on hepatocellular carcinoma cells

**DOI:** 10.1038/s41598-023-40809-y

**Published:** 2023-09-01

**Authors:** Zehra Omeroglu Ulu, Nurdan Sena Degirmenci, Zeynep Busra Bolat, Fikrettin Sahin

**Affiliations:** 1 Department of Genetics and Bioengineering, Faculty of Engineering, Yeditepe University, Kayısdagi Cad., Atasehir, 34755 Istanbul, Turkey; 2 Department of Molecular Biology and Genetics, Faculty of Natural Sciences, Istanbul University, 34134 Istanbul, Turkey; 3 Department of Molecular Biology and Genetics, Faculty of Engineering and Natural Sciences, Istanbul Sabahattin Zaim University, 34303 Istanbul, Turkey; 4 Molecular Biology and Genetics Department, Hamidiye Institute of Health Sciences, University of Health Sciences-Turkey, 34668 Istanbul, Turkey; 5 Experimental Medicine Research and Application Center, University of Health Sciences-Turkey, 34662 Istanbul, Turkey

**Keywords:** Cancer genomics, Gene ontology, Hepatocellular carcinoma, Cancer, Computational biology and bioinformatics

## Abstract

Hepatocellular carcinoma (HCC) is a leading cause of cancer-related death in the world. Poor prognosis of HCC patients is a major issue, thus, better treatment options for patients are required. Curcumin (Cur), hydrophobic polyphenol of the plant turmeric, shows anti-proliferative, apoptotic, and anti-oxidative properties. Boron is a trace element which is essential part of human nutrition. Sodium pentaborate pentahydrate (NaB), a boron derivative, is an effective agent against cancer. In the current study, we performed in vitro experiments and transcriptome analysis to determine the response of NaB, Cur, piperine (Pip) and their combination in two different HCC cell lines, HepG2 and Hep3B. NaB and Cur induced cytotoxicity in a dose and time dependent manner in HepG2 and Hep3B, whereas Pip showed no significant toxic effect. Synergistic effect of combined treatment with NaB, Cur and Pip on HCC cells was observed on cytotoxicity, apoptosis and cell cycle assay. Following in vitro studies, we performed RNA-seq transcriptome analysis on NaB, Cur and Pip and their combination on HepG2 and Hep3B cells. Transcriptome analysis reveals combined treatment of NaB, Cur and Pip induces anti-cancer activity in both of HCC cells.

## Introduction

HCC is a leading cause of cancer mortality worldwide^1^. Each year, approximately 800,000 new cases of primary liver cancer are reported, and a staggering 90% of these cases are attributed to HCC. The two-year survival rate in the US is less than 50%, and the five-year survival rate is only 10%^2,3^. HCC treatment line includes surgical resection, radiotherapy, chemotherapy and immunotherapy. Despite the achievements on the current treatment strategies, high recurrence rate and poor prognosis of HCC patients is still an issue^4^. Thus, new strategies are necessary for the diagnosis and treatment of HCC. Boron is a rare element showing biological functions in different organisms. Boron derivatives show therapeutic potential in different cancers such as hepatocellular carcinoma^5^, multiple myeloma^6^, breast^7^ and small-cell lung cancer^8^. Bortezomib is the only boron containing chemotherapeutic drug approved for clinical use however, drug resistance rapidly develops in bortezomib treatment^9^. NaB is another boron derivative that is known to be an effective agent in obesity^10^, wound healing^11^, cryopreservation^12^ and cancer^13^. NaB showed metabolic reprogramming related to SIRT3 activity in Hep3B cell lines^14^.

Cur is the main hydrophobic polyphenol in the rhizome of the plant turmeric. Cur is known to have anti-inflammatory, antioxidant, anti-microbial and anti-cancer properties^15,16^. Cur induces apoptosis and autophagy in HCC. Despite curcumin’s significant therapeutic value, the delivery of Cur is still an important issue as it shows photodegradation, short half-life, low bioavailability, and pharmacological activity^17^. Pip, isolated from black and long peppers, is an important plant alkaloid. It is known to improve curcumin’s poor bioavailability by reducing the rate of its metabolic breakdown^18^. Combined treatment of Pip and Cur has been reported to enhance the anti-cancer effect in colorectal^19^, leukemia^20^ and breast^21^ cancer cells. Furthermore, in vivo studies show that Cur and Pip combination treatment induced HCC in rats^22^.

RNA-seq is a leading and high-throughput sequencing approach to provide insight into the transcriptome of a cell. Compared to previous Sanger sequencing- and microarray-based methods, RNA-Seq facilitates the discovery of novel transcripts, identification of alternatively spliced genes, and detection of allele-specific expression beyond quantifying gene expression^23^. Recent advances in the RNA-Seq workflow, from sample preparation to library construction to data analysis, have enabled researchers to further elucidate the functional complexity of the transcription. RNA-seq data analysis typically includes several steps: quality control, alignment, counting and normalization of the sequenced reads, and differential expression (DE) analysis^24,25^.

**Figure 1:**
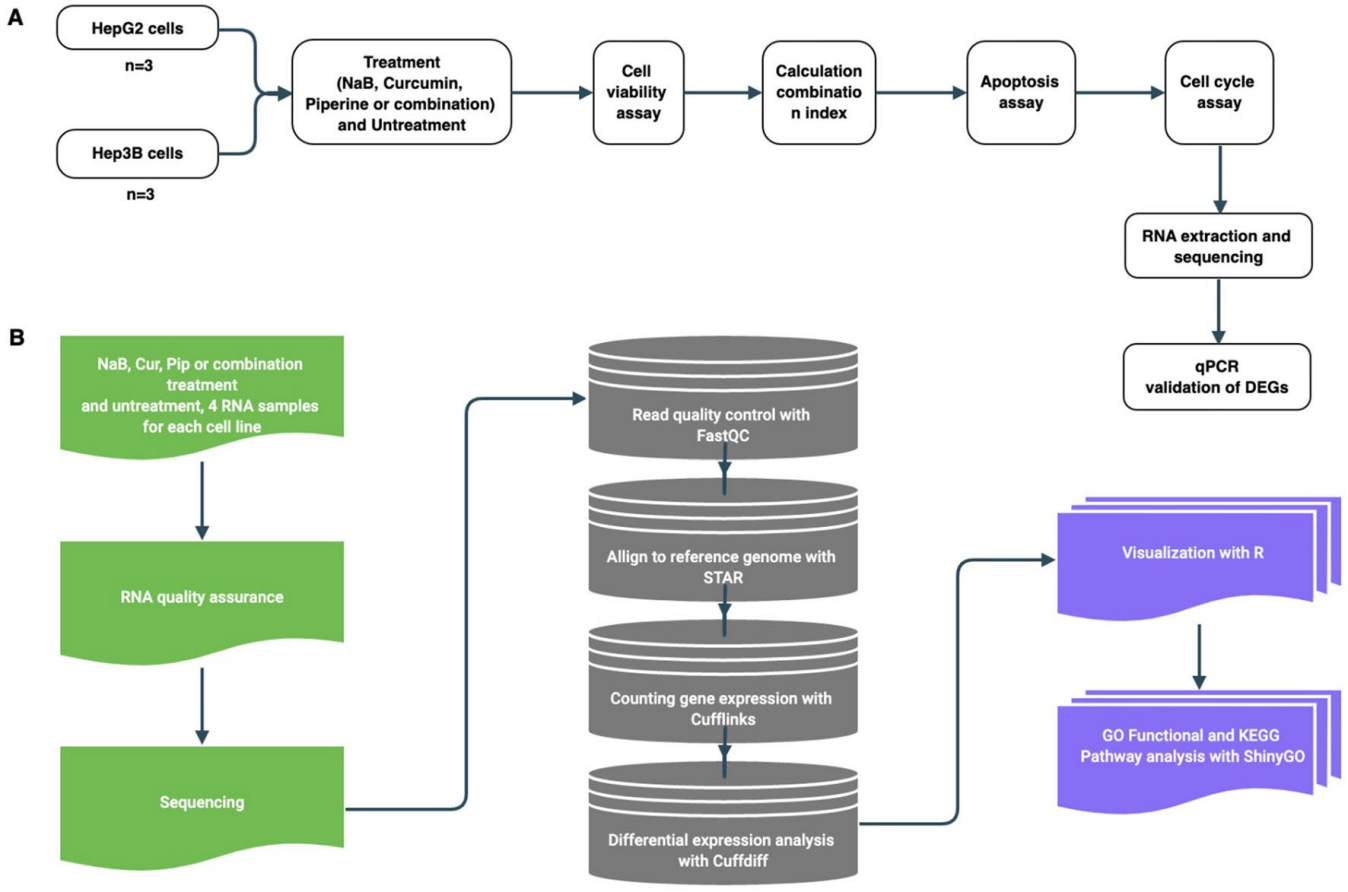
Study design: (**A**) Diagram representation of the experimental workflow. (**B**) The RNA-seq and bioinformatic analysis workflow.

In our study, RNA-seq transcriptome analysis was performed to examine the possible anti-cancer mechanisms of NaB, Cur and Pip combination exerting synergistic effect on HCC cells. First, cell viability of HepG2 and Hep3B cells were investigated under the treatment of NaB, Cur and Pip and their combination treatment. Next, apoptotic cell death and cell cycle analysis were determined for HepG2 and Hep3B cells treated with NaB alone or in combination Cur and Pip or in combination NaB, Cur and Pip (Fig. 1A). The in vitro studies were followed by RNA-seq transcriptome analysis to determine the synergistic effect on gene expression profiling and we verified the anti-cancer effect on functional and pathway analysis (Fig. 1B).

## Results

### Combination treatment of NaB, Cur and Pip inhibits growth of HCC cells

**Figure 2:**
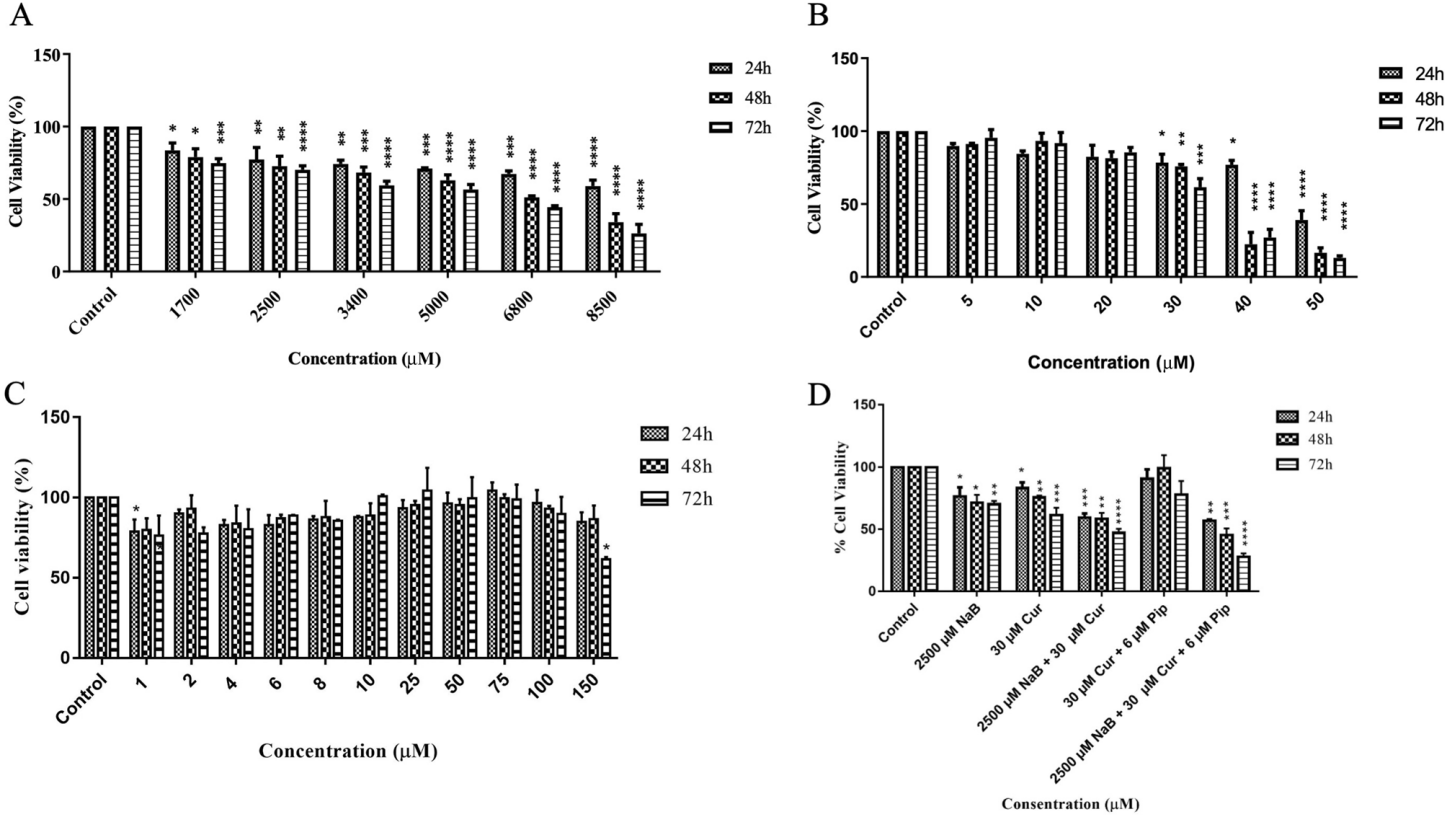
Effect of NaB, Cur, Pip and their combination treatment on cell viability of HepG2 cells. HepG2 Cells were incubated with (**A**) (1700–8500 µM) NaB, (**B**) (5–50) µM of Cur, (**C**) (1–150) µM of Pip and (**D**) combinations of NaB, Cur and Pip for 24 h, 48 h and 72 h. The cell viability was determined by MTS assay. Data represent the mean ± SD of independent experiments (n = 3) and statistical significance was assessed by one way ANOVA.

**Figure 3:**
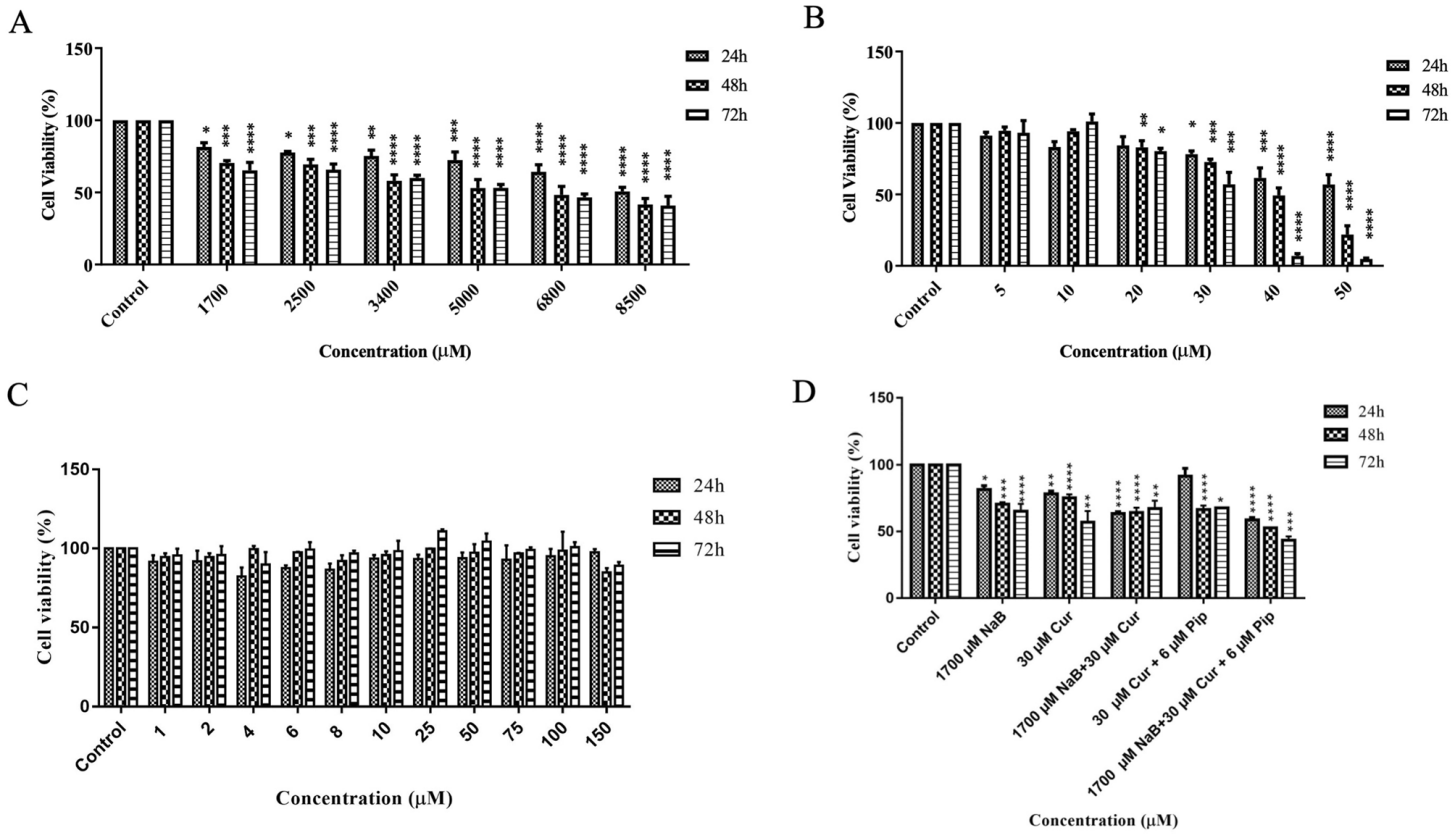
Effect of NaB, Cur, Pip and their combination treatment on cell viability of Hep3B cells. Hep3B Cells were incubated with (**A**) (1700–8500 µM) NaB, (**B**) (5–50) µM of Cur, (**C**) (1–150) µM of Pip and (**D**) combinations of NaB, Cur and Pip for 24 h, 48 h and 72 h. The cell viability was determined by MTS assay. Data represent the mean ± SD of independent experiments (n = 3) and statistical significance was assessed by one way ANOVA.

To assess the drug interaction effect of NaB and Cur together, HepG2 and Hep3B cells were exposed to different concentrations of both drugs alone and in combination for 24, 48 and 72 h. NaB and Cur effectively inhibited growth of HepG2 cells in time and dose-dependent manner (Fig. 2A,B), while Pip treated cells showed no toxic effect except the highest dose (Fig. 2C). Combination treatment of NaB, Cur and Pip demonstrated significant cytotoxicity in time-dependent manner in HepG2 cells (Fig. 2D). Similarly, NaB and Cur effectively inhibited growth of Hep3B cells in time and dose-dependent manner (Fig. 3A,B). Pip treated cells showed no toxic effect (Fig. 3C) and combination treatment of NaB, Cur and Pip demonstrated significant cytotoxicity in time-dependent manner in Hep3B cells (Fig. 3D). HUVEC showed less toxicity compared to HCC cells when treated with a combination of NaB, Cur and Pip (Fig. S1).

The IC_50_ values of NaB and Cur against HepG2 cells for 48 h are 7664.5 µM and 44.8 µM, respectively. While the IC_50_ values of NaB and Cur against Hep3B cells are 6561 µM and 41.4 µM, respectively. Furthermore, combination of NaB and Cur was considered as a drug, as Pip improves the therapeutic effect and bioavailability of Cur. The CI value for NaB and Cur combination of 2.5 mM NaB and 30 µM Cur in HepG2 cells were calculated as 0.98. 1.7 mM NaB and 30 µM Cur combination treatment in Hep3B cells showed combination index (CI) value of 0.97. This demonstrated consistent synergy (CI < 1) between NaB and Cur, validate our selection criteria (Table S1).

### Combination treatment of NaB, Cur and Pip induce apoptosis and arrest the progression of cell cycle in HCC

**Figure 4:**
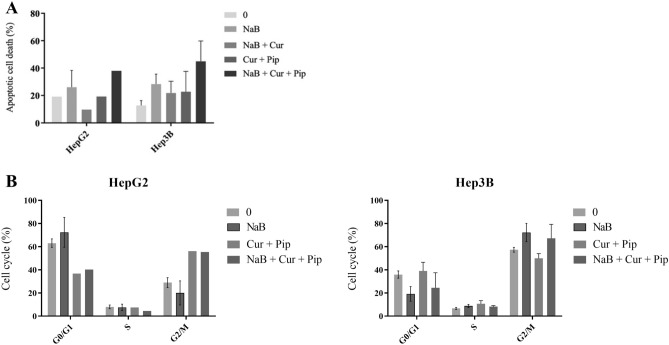
(**A**) The percentage of cells undergoing apoptotic cell death in HepG2 under 2500 µM NaB, 30 µM Cur and 6 µM Pip treatment, and Hep3B cells under 1700 µM NaB, treatment at 48 h. (**B**) Effect of NaB, Cur and Pip on cell cycle progression in HCC cell lines. Percentage of cell cycle profiles in HepG2 and Hep3B cells under NaB, Cur and Pip treatment at 48 h was analyzed by flow cytometry.

Annexin V/PI apoptosis assay was performed to determine the apoptotic cell death percentage (representing the early and late apoptosis) after treating HCC cells with combination treatment of NaB, Cur and Pip. Autofluorescence for each cell line was established with an unstained control. Before each reading compensation procedure is performed for untreated samples using propidium iodide (PI) only, FITC only and FITC-PI double labelled standards. Our results showed that NaB, Cur and Pip combined treatment induced apoptosis in HepG2 and Hep3B cells, showing nearly 40% apoptotic cell death (Fig. 4A). To investigate the mechanisms underlying changes in cell cycle progression of HCC cells when treated with combination of NaB, Cur and Pip flow cytometry was used. As shown in Fig. 4B, our results suggest that combination of NaB, Cur and Pip inhibited cellular proliferation of HCC cell lines, HepG2 and Hep3B via arrested G0-G1 phase of the cell cycle.

### RNA-seq transcriptome analysis of HCC under combination therapy of NaB, Cur and Pip

**Figure 5:**
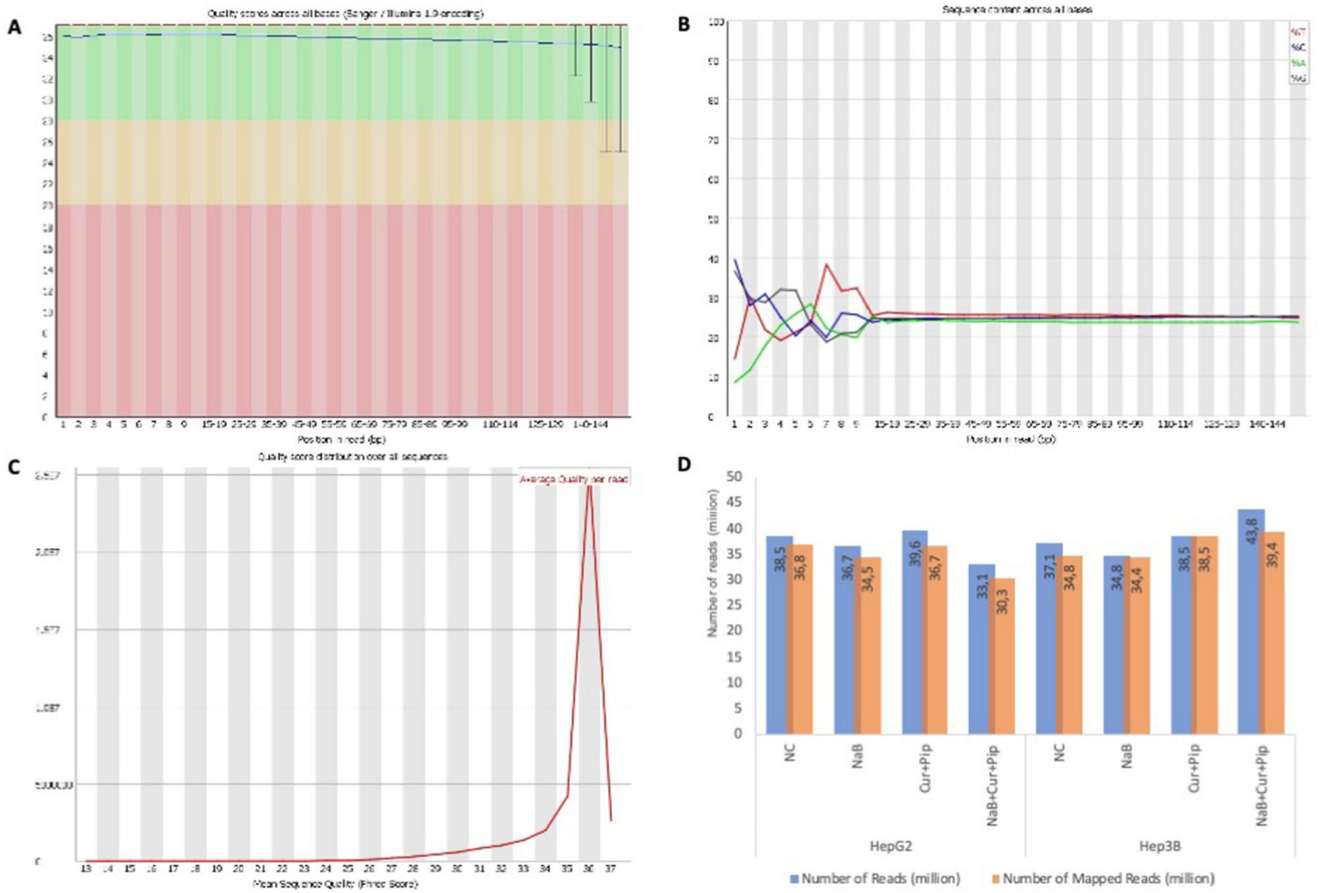
Quality control and statistics of the sequenced reads. (**A**) Distribution of quality scores by base pair. (**B**) The nucleotide content (A, T, C and G) in sequenced reads. (**C**) Distribution of the mean quality score by read. (**D**) The number of reads and mapped reads in HepG2 and Hep3B cells RNA-seq data.

**Figure 6:**
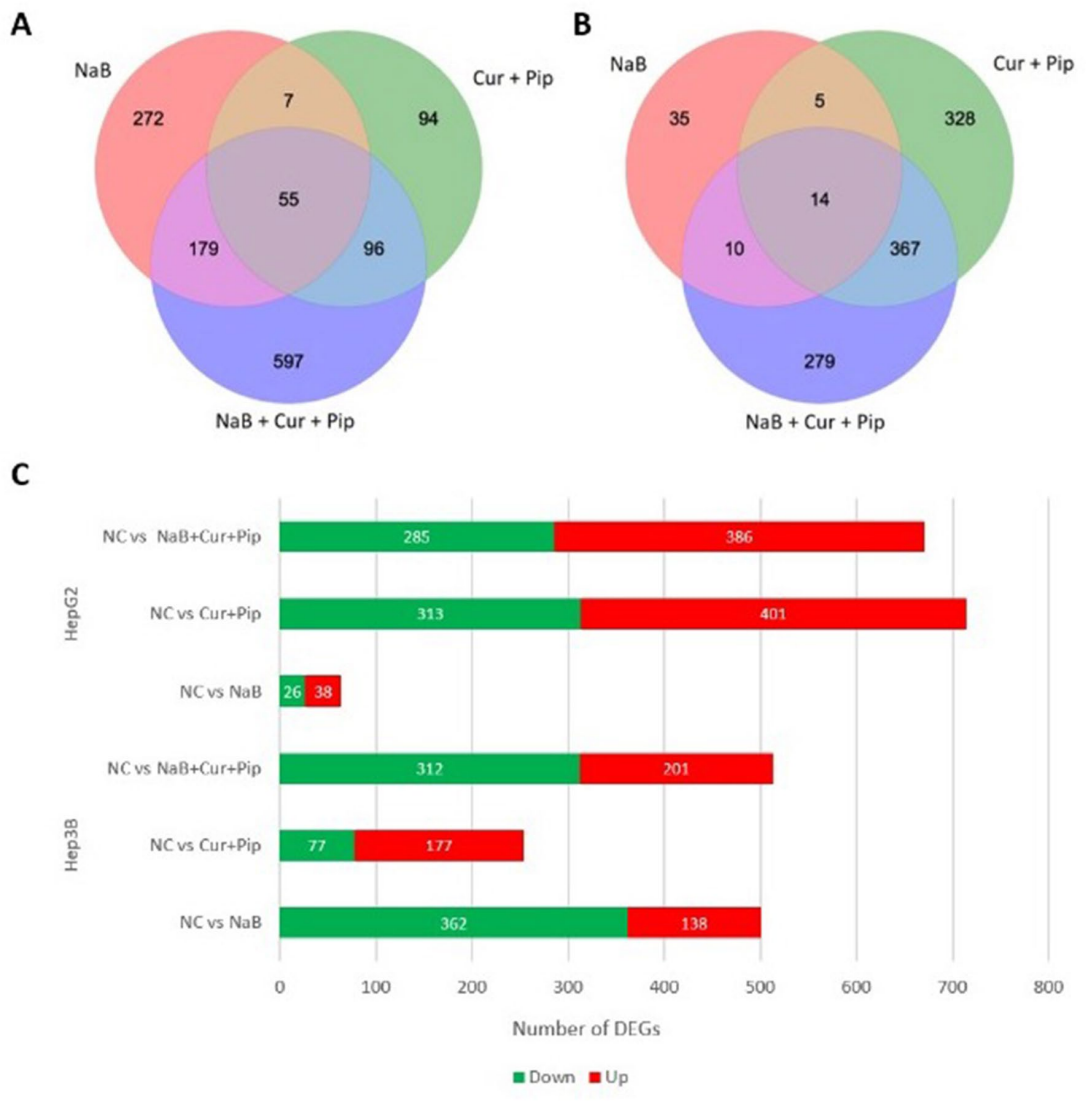
The overview of DEGs identified by RNA-seq analysis. (**A**) The Venn diagram shows NaB, Cur and Pip and their combination DEGs in HepG2 cells. (**B**) The Venn diagram shows NaB, Cur and Pip and their combination DEGs in Hep3B cells. (**C**) The number of DE up-regulated (red) and down-regulated (green) genes in HepG2 and Hep3B cells.

We performed RNA-seq transcriptome analysis on NaB, Cur and Pip and their combinations on HCC cells. The quality control of the sequencing read files with FastQC and the quality of the reads is shown in Fig. 5. We carried out the mapping of reads using STAR**.** The read and mapped number of HepG2 and Hep3B cells treated with NaB alone, Cur and Pip, combination of all three and untreated groups were shown in Fig. 6. The BAM output files were processed with Cufflinks for the detection of expression counts for downstream analysis. We evaluated the differentially expressed genes (DEGs) after FPKM normalization, and found total of 3143 genes (P < 0.01 and log_2_FC(fold change) > |1.5|) at Cuffdiff, and at least 64 counts in any sample). In Fig. S2 was shown the dispersion plot and the scatter plot of distribution of all of the expressed genes in HepG2 and Hep3B data. We showed number of up-down regulated and Venn diagrams analysis of common genes after NaB, Cur, Pip or combination treatment (P < 0.01, log_2_FC(fold change) > |1.5|) in Fig. 6.

**Figure 7:**
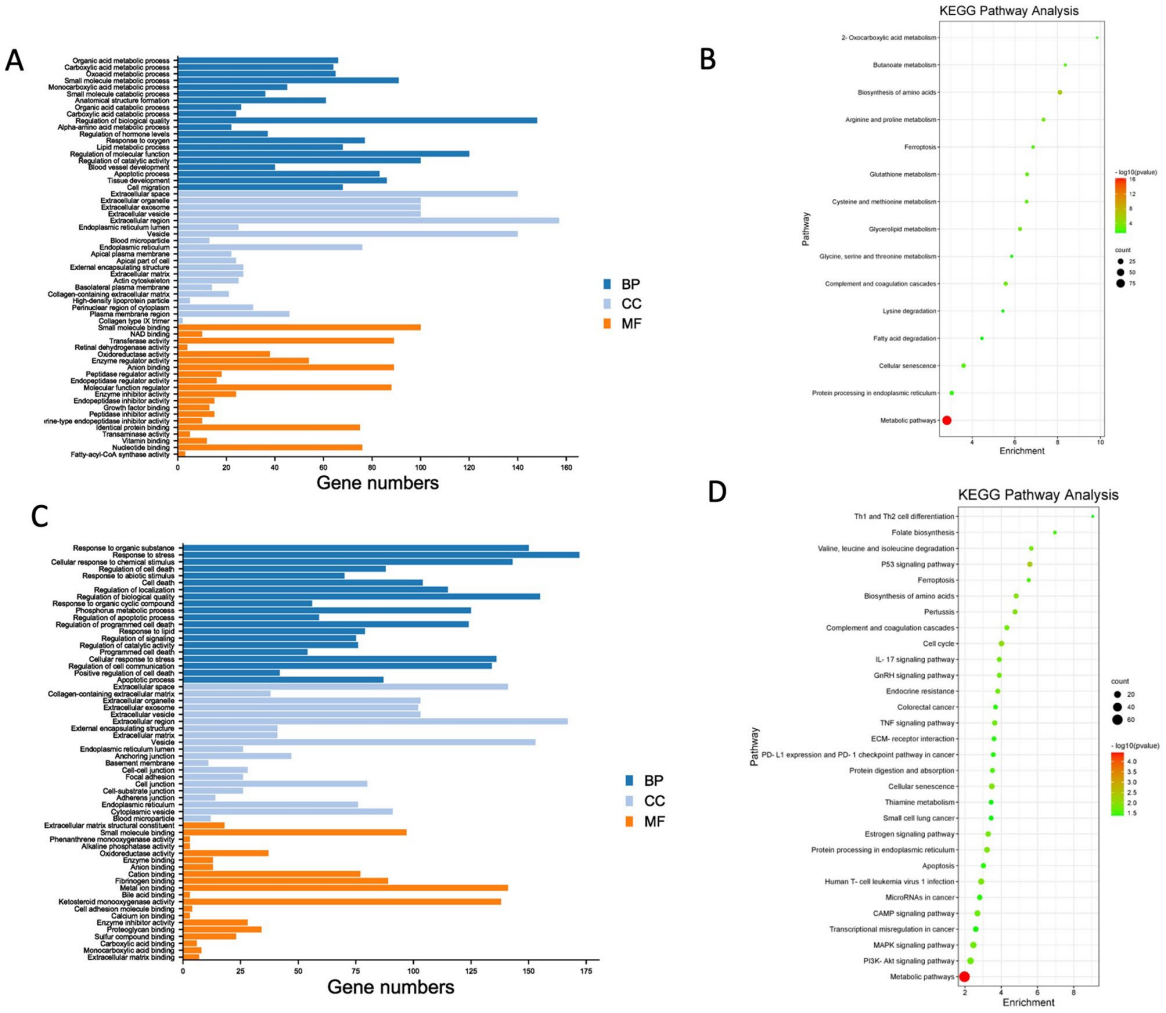
The top 20 Gene Ontology (GO) [Biological process (BP); Cellular component (CC), Molecular function (MF)] and KEGG pathway results for combination DEGs. (**A**) The GO results of combination DEGs in HepG2 cells. (**B**) The KEGG pathway results of combination DEGs in HepG2 cells. (**C**) The GO results of combination DEGs in Hep3B cells. (**D**) The KEGG pathway results of combination DEGs in Hep3B cells**.**

The top 20 enriched Gene Ontology (GO) terms in the categories and KEGG pathway terms for DEGs in HepG2 and Hep3B after combination treatment were shown in Fig. 7A–D, respectively. The enriched functions of the DEGs in both of HCC cells include apoptotic process, regulation of biological quality and catalytic activity and related cell death terms. Also, the results and KEGG pathway enrichment analysis showed that the genes regulated by combination therapy were significantly involved in cancer-related pathways such as apoptosis, ferroptosis, cellular senescence, cell cycle, p53 signaling pathway, MAPK signaling pathway, IL-7 signaling pathway and TNF signaling pathway.

**Figure 8:**
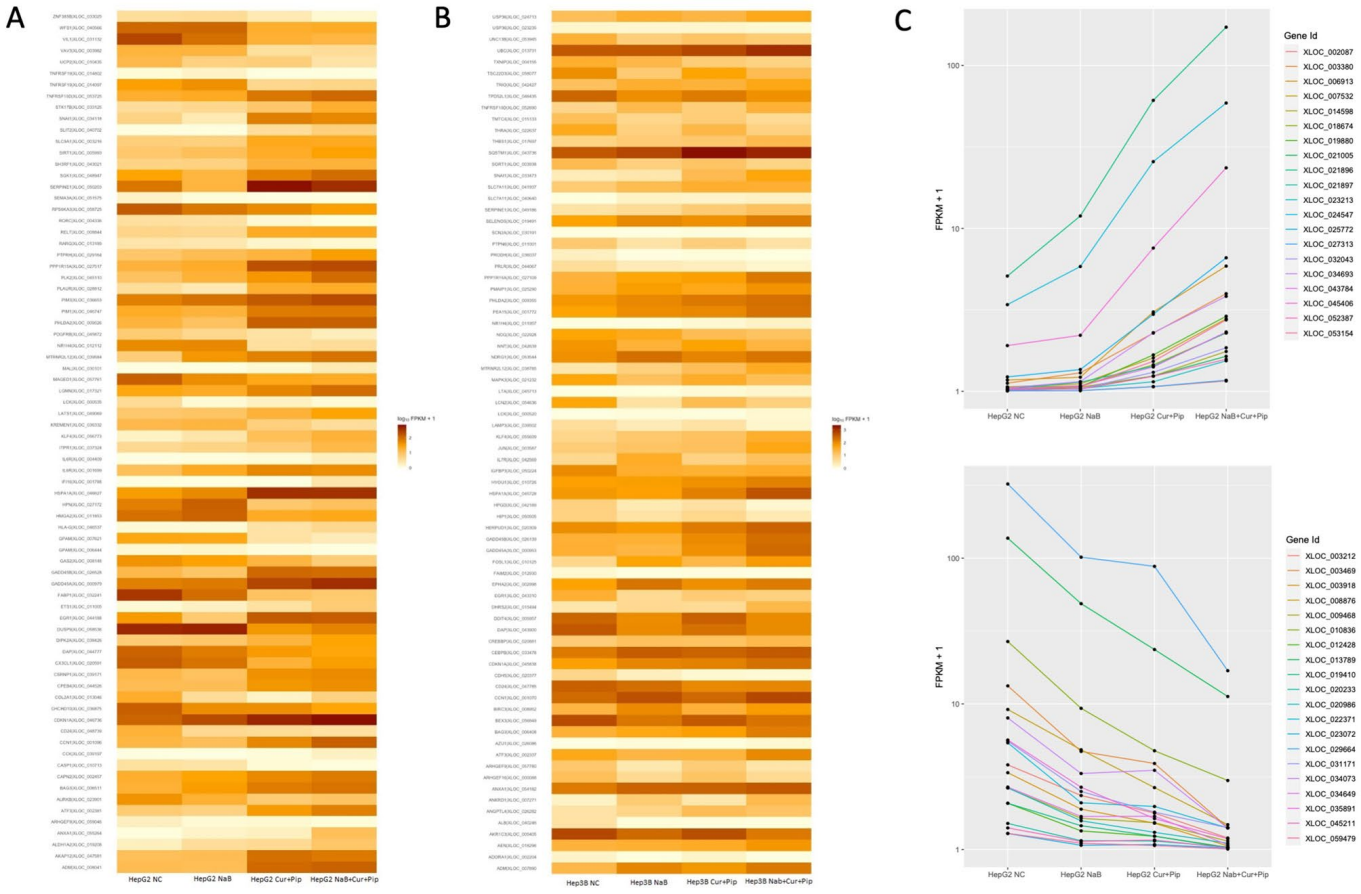
Heatmaps of combination DEGs in GO BP apoptotic process term and some similar effected combination DEGs. (**A**) The heatmap of DEGs after combination treatment in HepG2 cells. (**B**) The heatmap of DEGs after combination treatment in Hep3B cells. (**C**) The expression profiling of some synergistically affected DEGs in combination treatment.

A heatmaps of the genes associated with the apoptotic process is shown in Fig. 8A and B. The gene expression patterns from untreated cells to NaB or Cur and Pip treatment were similar than that from untreated cells to combination treatment within the GO apoptosis process term. Furthermore, some similar synergistic effected genes to a provided expression profiles after combination treatment were shown in Fig. 8C.

### Validation of the RNA‐seq results using qRT-PCR

To validate the results of DEGs (p-value < 0.05) in transcriptome sequencing, *GADD45A* (Growth Arrest and DNA Damage Inducible Alpha), *BBC3* (BCL2 Binding Component 3) *CDKN1A* (Cyclin Dependent Kinase Inhibitor 1A), *PMAIP1* (Phorbol-12-Myristate-13-Acetate-Induced Protein 1) and *SERPINE1* (plasminogen activator inhibitor-1, PAI-1) which are apoptotic cell death related genes were selected for qRT-PCR. Compared to the NaB or Cur alone treated HepG2 cells, *GADD45A*, *BBC3*, *PMAIP1* and *SERPINE1* were up-regulated in combination treated groups (Fig. S3A). On the other hand, the combination treatment of NaB, Cur and Pip up-regulate *GADD45A*, *CDKN1A*, *PMAIP1* and *SERPINE1* genes in Hep3B cells compared to NaB or Cur alone treated groups (Fig. S3B).

## Discussion

HCC is a common type of cancer that is caused by cirrhosis of the liver^26^. It is the most malignant primary human liver cancer, which is the sixth most common diagnosed cancer type and the third cause of cancer-related deaths in the world^27,28^. First line therapy methods include surgical, radiotherapy and chemotherapy^29^. However, poor therapeutic profile and high recurrence rate leads for the need of new strategy approaches and effective anti-cancer agents in treatment of HCC.

Various new agents besides chemotherapeutic drugs for cancer treatments have recently been discovered and boron-based agents are one of the forefront agents^27^. Studies have shown that boron derivatives have lethal effect on cancer cells by inhibiting cell division and increasing apoptosis. It can be used as anti-cancer agents in various cancers, such as the breast, lung, and prostate^30^. Previously, we have demonstrated that sodium perborate causes anti-proliferative and apoptotic effect in HepG2 and Hep3B cell lines^5^. NaB, also a boron derivative, is an effective agent cancer and shows SIRT3 activity in HCC Hep3B cell lines^14^. Although these boron derivative molecules show anti-cancer effect on HCC cells, high killing doses are needed. Thus, combination therapy is applied by combining two or more anti-cancer agents to increase the effectiveness of the agents on cancer cells and create a synergistic effect. It is also a treatment that can develop more effective cancer drugs^31^.

Cur shows anti-proliferative, apoptotic, and anti-oxidative properties on many cancers, along with its pharmacological effects in many cancer types^32^. However, limitations on pharmacological activity of Cur along with its low bioavailability and short half-life^17^, is a major issue to be used as anti-cancer agent alone. Studies show that Pip solves this problem by improving Cur’s poor bioavailability^18^ and enhances anti-cancer effect in many cancer cells^19,20^. Furthermore, Cur can be used alone or in combination with other drugs, targets signaling pathways that trigger cancer development^33^. Anti-cancer studies show that Cur composes a synergistic effect with different anti-cancer drugs such as the PI3K inhibitor^34^ and erlotinib^35^. Cur and 5-fluorouracil showed synergistic effect on HCC cells, leading to an increase in efficacy of 5-fluorouracil and decrease of concentration usage of 5-fluorouracil^36^. When drugs used in chemotherapy are applied as monotherapy on cancer cells, both high doses are applied, and their effects on cells are weak. For this reason, combination studies are essential in cancer studies.

In this study, we aimed to create a synergistic effect in HCC HepG2 and Hep3B cell lines by combination treatment of NaB, Cur and Pip. The current study investigated the cytotoxic, cell cycle arrest and apoptotic cell death effects of NaB, Cur and Pip in HCC cells, alone or in combination therapy. In addition, RNA-seq analyses were used to explore the molecular mechanism of NaB, Cur and Pip on HCC cells, followed by RT-qPCR to perform validation studies.

To characterize the cytotoxic effect of NaB, Cur and Pip, we first investigated the cell viability of HepG2 and Hep3B cells treated with either alone or in combination of NaB, Cur and Pip. Our cytotoxicity results show that HCC cells treated with NaB, Cur or Pip alone demonstrated a decrease in cell viability in time and dose dependent matter. Recent studies show IC_50_ concentration of HepG2 cells with boric acid and sodium perborate was determined as 24 mM and 1.13 mM^37^. Our results showed that NaB treated alone HepG2 and Hep3B cells had an IC_50_ value of 7.6 mM and 6.5 mM.

In combination therapy the effective dose was decreased to 2.5 mM and 1.7 mM for HepG2 and Hep3B cells, respectively. Thus, it was observed that low concentrations of NaB, Cur and Pip formed a synergistic effect, statistically significantly reduced the cell viability of HepG2 and Hep3B cells according to the time and dose manner. When the CI value is CI < 1, it shows a synergy in combination treatment^38,39^, thus our results show synergistic effect as the CI value for both HCC cells are less than one. Therefore, in this study we have shown that combination therapy NaB, Cur or Pip increased the effectiveness of the agents on cancer cells and create a synergistic effect by lowering the dosage of NaB.

To determine the effect of combination treatment of NaB, Cur and Pip on healthy cells, HUVEC was chosen as model cell line. Sodium perborate did not cause a significant decrease in cell viability of HUVEC cells compared to HCC cells^5^ and our results are in consistency with literature as HUVEC showed less toxicity compared to HCC cells when treated with the same combination dosage of NaB, Cur and Pip.

Next, we investigated the apoptotic cell death and cell cycle progression in HCC cells when treated with NaB alone or combined with Cur and Pip. The combination study revealed that the apoptotic effect on cancer cells increased compared to alone treatment of NaB in HCC cells. Thus, combination treatment of NaB, Cur and Pip showed synergistic effect in apoptotic cell death of both HCC cells. Sodium perborate arrested cell cycle of HepG2 and Hep3B cells in G0/G1 phase^5^. Similarly, our results show that combination of NaB, Cur and Pip arrested HCC cells at G0-G1 phase.

To further explore the molecular mechanism of effect of NaB, Cur and Pip treatment and their combination treatment on two different HCC cells, we performed the gene expression profiling by RNA-seq on HCC cells. To identify genes regulated by NaB, Cur and Pip and their combination treatment, determine synergistic effect on gene regulation and further elucidate the molecular mechanism by which NaB, Cur and Pip combination inhibits HCC cell proliferation, transcriptome analysis were carried out on NaB, Cur and Pip, their combination treatment and non-treatment of HCC cells. A total of 671 and 513 DEGs were observed in NaB, Cur and Pip combination treatment of HepG2 and Hep3B, respectively. These DEGs were further analyzed by GO functional enrichment which was annotated into biological process (BP), cellular component (CC), molecular function (MF) classifications and KEGG pathway enrichment analysis. The top 20 GO enriched classes and KEGG pathway results of combination DEGs of HepG2 and Hep3B cells are shown in Fig. 7. The GO results indicated that combination DEGs were significantly involved in apoptotic process GO term in both HCC cell lines and KEGG results showed that combination DEGs were significantly involved in cancer-related different signaling pathways such as apoptosis, ferroptosis, cellular senescence, cell cycle, p53 signaling pathway, MAPK signaling pathway, IL-7 signaling pathway and TNF signaling pathway. According to a previous study, the results of the microarray analysis of boric acid treated HepG2 cells showed that boric acid induced cell cycle, DNA replication and p53 signaling pathways^37^. Treatment of sodium perborate on HepG2 and Hep3B cells induced apoptosis and cell cycle related terms in RNA-seq GO and KEGG analysis^5^, which is consistent with our results. Besides, an array-based study of Cur treated five HCC cell lines showed that up-regulated and down-regulated DEGs enriched in TNF signaling pathway, NF-kappa B signaling pathway, TGF-beta signaling pathway and AMPK signaling pathway^40^. Thus, our results were in consistent with literature (Fig. 7).

*SERPINE1* plays an important role in p53 pathway^41^ and *PMAIP1* gene also inhibits proliferation and has a role in induction of apoptosis^42^. Previously we have shown that sodium perborate increases *SERPINE1* and *PMAIP1* genes expression in both HCC cells^5^. Similarly, our RNA-seq analysis of combination treatment of NaB, Cur and Pip in HepG2 and Hep3B cells showed increase in *SERPINE1 and PMAIP1* genes expression. Furthermore, our results demonstrate an increase in *GADD45A* gene expression in HepG2 and Hep3B cells. *GADD45A* gene plays a essential roles in regulation of many cellular functions including DNA repair, cell cycle arrest control, pro-apoptotic activity and tumor growth suppression^43^. Besides, *BBC3* gene shows a pro-apoptotic gene activity^44^ and raises in HepG2 cells with treatment combination of NaB, Cur and Pip compared to NaB or Cur alone treated HepG2 cells. Also, in combination of NaB, Cur and Pip treated Hep3B cells, *CDKN1A* gene which has a role in regulation of cell cycle process^45^ showed upregulated compared to NaB or Cur alone treated Hep3B cells. We have validated the accuracy of the RNA-seq data using RT-qPCR analyses.

In summary, our results demonstrated that combination treatment of NaB, Cur and Pip had synergistic effect in HepG2 and Hep3B cell lines. The combination of NaB, Cur and Pip inhibits cell proliferation and induces apoptosis in HCC cells compared to NaB or Cur alone treated HCC cells. We further examined the gene expression pattern of NaB, Cur and Pip treatment and their combination treatment on two different HCC cell lines using RNA-seq. According to these results, the expression level of some DEGs demonstrated the synergistic effect in combination of NaB, Cur and Pip treated HCC cells compared to NaB or Cur alone treated HCC cells and NaB, Cur and Pip affected DEGs regulated several anti-cancer mechanisms. These findings suggested that combination of NaB, Cur and Pip may be a promising drug in the clinical treatment of HCC.

## Materials and methods

### Chemicals and reagents

NaB was purchased from National Boron Research Institute-BOREN (Ankara, Turkey). NaB stocks were prepared as 10 mg/mL in completed media using vortex and all solutions were filtered with a 0.22 μm filter before use. Cur and Pip were purchased from Sigma-Aldrich (St. Louis, MO, USA) and resuspended in dimethyl sulfoxide (DMSO) (Sigma-Aldrich, St Louis, MO) to prepare a stock solution of 50 mM and 150 mM, respectively. The main stock solution was filtered through a 0.22 μm filter and kept at − 20 °C for long time storage.

MTS reagent ([3-(4,5-dimethylthiazol-2-yl)-5- (3-carboxymethoxyphenyl)-2-(4-sulfophenyl)-2H-tetrazolium) was purchased from Promega (Southampton, UK). Annexin V-FITC Apoptosis Detection Kit was purchased from Roche (Germany). RNase A and PI were obtained from Sigma-Aldrich (Germany). Nonidet P-40 was obtained from AppliChem (Germany). RNeasy Mini kit, Sensiscript Reverse Transcription PCR Kit and QuantiTect SYBR Green PCR kit were supplied by Qiagen (United States).

### Cell culture

Two different human HCC cell lines, HepG2 (HB-8065, ATCC), Hep3B (HB-8064, ATCC) were cultured in DMEM media supplemented with 10% fetal bovine serum (FBS) and 100 units/mL of penicillin, 100 μg/mL of streptomycin and amphotericin (1% PSA). HUVEC (CRL-1730) cell line, selected as normal cells, was maintained in DMEM media completed with 10% FBS and 1% PSA. All cells were incubated at 37 °C in a humidified atmosphere with 5% CO_2_.

### Cytotoxicity assay

The effect of NaB, Cur and Pip and their combination on both HCC and HUVEC cell lines were investigated using cell proliferation reagent MTS reagent assay. HepG2 (5 × 10^3^ cells/well), Hep3B (5 × 10^3^ cells/well) and HUVEC (5 × 10^3^ cells/well) cells were seeded on 96 well plates. Cells were treated with (1700–8500) µM of NaB, (5–50) µM of Cur, (1–150) µM of Pip or combination of all three. At each 24 h interval, cells were incubated with appropriate MTS solution mixture for 2 h and absorbance was measured at 490 nm using a microplate spectrophotometer (Bio-tek ELx800, USA). Cell viability (%) was determined by setting non-treated control cells to 100%.

### Combination index calculation

The multiple drug effect analysis of Chou and Talalay^38,39^, which is based on the median-effect principle, was used to examine the nature of the interaction between NaB and Cur. Determination of the synergistic versus additive versus antagonistic cytotoxic effects of the combined treatment of cells with only NaB, only Cur and combination of NaB and Cur were assessed using the CI. The CI < 1, CI = 1 and CI > 1 indicate synergistic, additive and antagonistic effects, respectively. The CI was calculated using the following formula:

$${\text{CI}} = \left[ {\left( {{\text{D}}_{{1}} } \right) \, /\left( {{\text{D}}_{{\text{x}}} } \right)_{{1}} } \right] + \left[ {\left( {{\text{D}}_{{2}} } \right) \, /\left( {{\text{D}}_{{\text{x}}} } \right)_{{2}} } \right]$$

where: (D_x_)_1_, (D_x_)_2_ = the concentrations of NaB and Cur that induced a 50% inhibition of cell proliferation; (D_1_), (D_2_) = the concentrations of NaB and Cur in combination that also inhibited cell growth by 50%.

### Apoptosis and cell cycle assay

The relative percentage of apoptotic HepG2 and Hep3B cells and cell population percentages to NaB, Cur and Pip and their combination of all three were analyzed by Annexin V/PI assay kit (Roche, Cat. No. 11988549001) and cell cycle assay using flow cytometry. HepG2 cells were treated with 2500 µM NaB, 30 µM Cur and 6 µM Pip and their combinations, while Hep3B cells were treated with 1700 µM NaB, 30 µM Cur and 6 µM Pip and their combinations. For apoptotic cell death, pellets were collected after 48 h treatment, suspended in 1X annexin binding buffer and stained at room temperature for 15 min with 5 μL Annexin-V FITC and 5 μL PI according to manufacturer’s protocol. Samples were analyzed immediately by Becton Dickinson FACS calibur flow cytometer with 20,000 events. For cell cycle arrest analysis, cells were trypsinized, washed by 1 × PBS and fixed in 70% ethanol solution for 1 h at − 20 °C. After fixation, cells were washed twice with ice cold 1 × PBS and stained with a cell cycle solution containing 10 μg/mL RNase A, 0.01% non-idet P-40 and 8 μg/mL PI for 30 min. Guava easyCyte Flow Cytometer (Merck Millipore, Germany) was used to determine cell population percentages of samples.

### RNA preparation and sequencing

Total RNA was isolated from sampling for the HCC cell lines (HepG2 with 2500 µM NaB, 30 µM Cur + 6 µM Pip, 2500 µM NaB + 30 µM Cur + 6 µM Pip and without treatment for 48 h; Hep3B 1700 µM NaB, 30 µM Cur + 6 µM Pip, 1700 µM NaB + 30 µM Cur + 6 µM Pip and without treatment for 48 h) with RNeasy Mini RNA isolation kit (Qiagen; Valencia, CA, USA) in triplicate following the manufacturer’s protocol. We measured RNA concentration using the Nanodrop 2000 Spectrophotometer (Thermo scientific). RNA quality was confirmed to be more than 8.0 RIN value. RNA-seq libraries were conducted with the NEBNext Ultra II Directional RNA Library Prep Kit (New England Biolab, Ipswich, Massachusetts, USA), according to the manufacturer’s instructions. Finally, library quality was assessed on the Fragment Analyzer platform (Agilent Technologies, CA, USA). The resulting libraries were sequenced on the Illumina Novaseq 6000 System (Illumina, San Diego, USA) at Eurofins Genomics (Konstanz, Germany), resulting on approximately 30.3 to 43.8 million reads for each sample in a 2 × 150 bp pair-end.

### RNA-seq data and downstream analysis

Quality control and reads statistics were determined by FastQC program^46^. The clean reads were then mapped to the human reference genome (GRCh38) using STAR alignment tool under default settings^47^. Following the mapping, the transcripts assembly of each sample was done with Cufflinks program^48^. Then the output GTF files were merged into a single unified transcript using the Cuffmerge program. The merged transcripts were compared to the reference annotation using the Cuffcompare program. The FPKM (the fragments per kilobase of transcript per million fragments) method was used to estimate the expression levels of mRNAs in every sample. Differential expression analysis in different treatment groups were performed using Cuffdiff software, an absolute value of |log2 (fold change)| > |1.5| and a p-value < 0.01 were set as the filter criteria for significant differential expression. These results were then interpreted using ShinyGO^49^ for bioinformatics analysis to identify significant Gene Ontology (GO) functional terms BP, CC, MF and KEGG pathway^50^ terms among the subsets of NaB, Cur and Pip and their combination treated and untreated groups.

### Quantitative real-time-PCR (qRT-PCR) validation

RNA samples from NaB, Cur and Pip and their combination treated and untreated cells used for the RNA-seq were analyzed by qRT-PCR. First- strand cDNA was synthesized using Sensiscript Reverse Transcription PCR Kit qRT-PCR was performed on a CFX96 Real-Time PCR Detection System (Bio-Rad, Hercules, CA, USA) using QuantiTect SYBR Green PCR kit according to the manufacturer’s protocol. Primers specific for *GADD45A *(forward, 5′-AGAAGACCGAAAGGATGGATAAGG-3′; reverse, 5′-CGGCCCGGGTTGCTGAC-3′), *CDKNA1* (forward, 5′-AGGGGACAGCAGAGGAAG-3′; reverse, 5′-GCGTTTGGAGTGGTAGAAATCTG-3′), *BBC3* (forward 5′-ACGACCTCAACGCGCAGTA-3′; reverse, 5′-CTAGTTGGGCTCCATTTCTGG-3′), *PMAIP1* (forward, 5’-AGATGCCTGGGAAGAAG-3′; reverse 5′-AGTCCCCTCATGCAAGT-3′), *SERPINE1* (forward, 5′-CTTCCACCCGTCTCTCTG-3′; reverse, 5′-CTACCAGGCACACAAAAGC-3′). The relation mRNA expression levels were normalized to *GAPDH* (forward, 5′-GGAGCGAGATCCCTCCAAAAT-3′; reverse, 5′-GGCTGTTGTCATACTTCTCATGG-3′) gene and calculated using the 2^− ΔΔCt^ method.

### Statistical analysis

All data were analyzed statistically using one-way ANOVA or two-tailed Student’s t-test. GraphPad Prism (version 8.0.1) Software was used to perform statistical analysis and plotting the graph. The error bars represent standard error of the mean from at least three independent experiments. The significant level was set at *P ≤ 0.05, **P ≤ 0.01, ***P ≤ 0.001, ****P ≤ 0.0001.

### Supplementary Information

Supplementary Information.

## Data Availability

The sequencing data have been deposited at NCBI with BioProject Accession Numbers PRJNA848267. The transcript abundance file for each sample has been submitted and archived to the NCBI Gene Expression Omnibus (GEO; http://www.ncbi.nlm.nih.gov/geo/) under sample Accession Number GSE207439.
