# Supplementary material for: Synergistic anti-cancer effect of sodium pentaborate pentahydrate, curcumin and piperine on hepatocellular carcinoma cells
Source: Sci Rep. 2023 Sep 1;13:14404. doi: 10.1038/s41598-023-40809-y (PMC10474293; doi:10.1038/s41598-023-40809-y)
Supplement: Supplementary file 1 — Supplementary Information. [file 41598_2023_40809_MOESM1_ESM.docx]

**Supplementary Information**

**Figure S1.** Effect of NaB, Cur and Pip treatment on cell viability of HUVEC cells. Cells were incubated with two different concentrations (1700 or 2500 µM) of NaB with 30 µM Cur and 6 µM Pip for 48h. The cell viability was determined by MTS assay. Data represent the mean ± SD of independent experiments (n=3) and statistical significance was assessed by Student’s t-test.


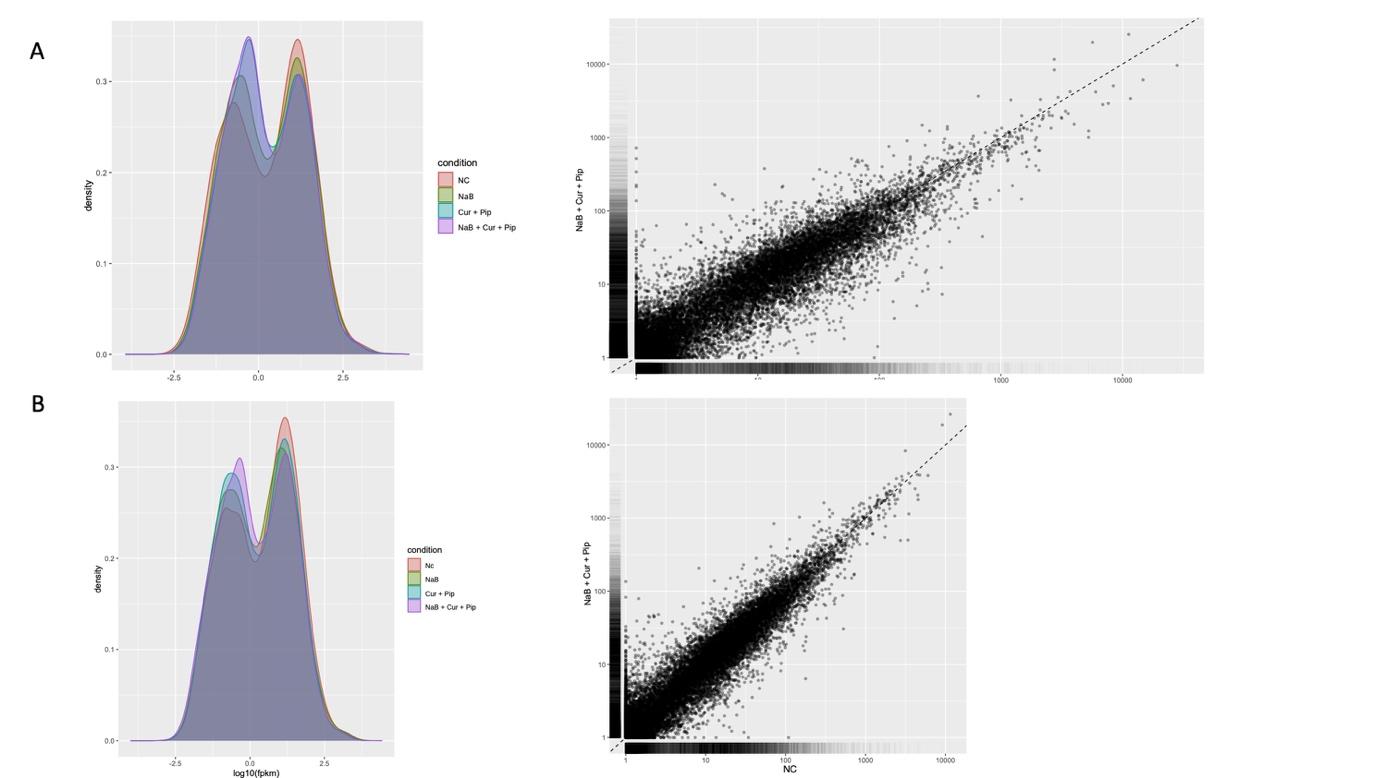


**Figure S2.** The dispersion plot and scatter plot show distribution of the expressed genes. **(A)** The dispersion and scatter plot of HepG2 cell data. **(B)** The dispersion and scatter plot of Hep3B cell data**.**


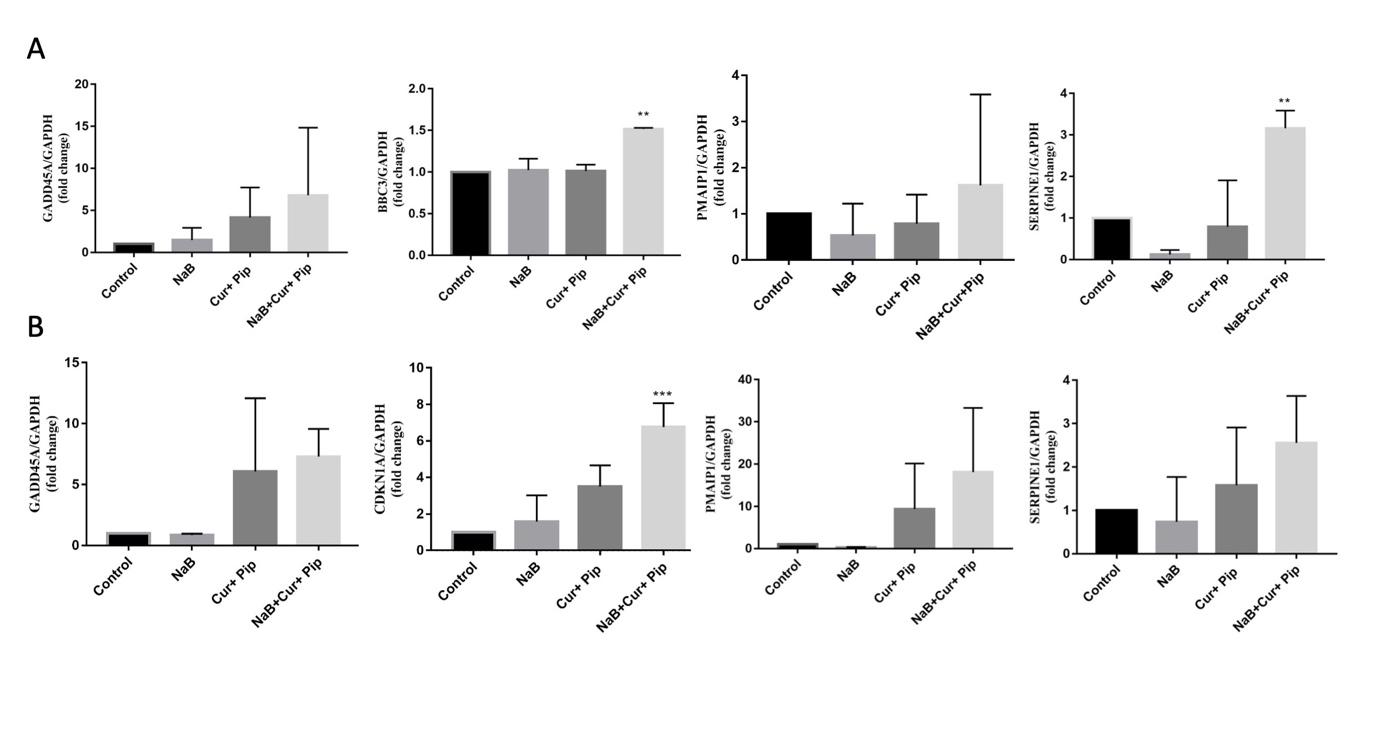


**Figure S3**: Illustration of qRT-PCR confirmation for RNA-seq. **(A)** Expression level of *GADD45A, BBC3, PMAIP1, SERPINE1* in HepG2 cells treated with NaB, Cur and Pip and their combination treated and untreated groups. **(B)** Expression level of *GADD45A, CDKN1A, PMAIP1, SERPINE1* in Hep3B cells treated with NaB, Cur and Pip and their combination treated and untreated groups.

**Table S1.** CI value of NaB and Cur combination treatment in HepG2 and Hep3B cells.

| **Cell type** | **Concentration of drug** | **CI value** |
| --- | --- | --- |
| HepG2 | 2500 µM NaB + 30 µM Cur | 0.98 |
| Hep3B | 1700 µM NaB + 30 µM Cur | 0.97 |
